# Analysis of Chemical Components and Blood‐Absorbed Components in Youjing Granules by UHPLC‐Q‐Orbitrap‐MS

**DOI:** 10.1002/ansa.70052

**Published:** 2025-10-29

**Authors:** Mingxin Guo, Jiaqi Zeng, Xuping Jiang, Wenjiao Zhu, Zhian Tang, Tieliang Ma

**Affiliations:** ^1^ The Affiliated Yixing Hospital of Jiangsu University Yixing Jiangsu China

**Keywords:** blood‐absorbed components, chemical components, pharmacodynamic material basis, ultra‐high‐performance liquid chromatography coupled with hybrid quadrupole‐orbitrap high‐resolution mass spectrometry (UHPLC‐Q‐orbitrap‐MS), Youjing Granules

## Abstract

This study investigates the chemical components of Youjing Granules (YG) and identifies blood‐absorbed components in rat serum following YG administration via gavage. Chemical components and blood‐absorbed components of YG were analysed and identified using ultra‐high‐performance liquid chromatography coupled with hybrid quadrupole‐orbitrap high‐resolution mass spectrometry (UHPLC‐Q‐orbitrap‐MS). Identification was achieved by comparing retention time, precise molecular weight, secondary MS fragments with literature data and reference substances. A total of 132 chemical components were identified and analysed from YG, primarily including flavonoids, prenol lipids, organooxygen compounds, isoflavonoids, steroids and steroid derivatives, as well as cinnamic acids and derivatives. Twenty‐four blood‐absorbed components were detected in serum, comprising 15 prototype components and 9 metabolites. The analysis of chemical components and blood‐absorbed components in YG using UPLC‐Q‐orbitrap‐MS technology provides a reference basis for further elucidating the pharmacodynamic material basis and mechanism of action of YG.

## 1 Introduction

Global male infertility rates show an upward trend, with sperm quality in men having decreased by approximately 50% over recent decades [1]. In industrialized countries, infertility affects 8%–12% of couples of childbearing ages, making it the third major threat to reproductive health after cardiovascular diseases and tumours [2, 3]. Primary causes of male infertility include physiological and genetic factors, behavioural lifestyles, environmental influences and socio‐demographic risks [3]. Given the complexity of etiological factors, treatment primarily focuses on etiological interventions, including pharmacotherapy, surgery and assisted reproductive technology (ART) [1]. However, these treatment methods present limitations and risks with unsatisfactory outcomes. For instance, ART carries risks such as high cost per cycle, low clinical pregnancy rates, complications like ovarian hyperstimulation syndrome and potential vertical transmission of epigenetic defects [4]. In contrast, traditional Chinese medicine (TCM) boasts a long history in treating male infertility. TCM emphasizes a holistic treatment approach that can effectively address functional disorders, demonstrating broad clinical applicability, improving reproductive function, laying a physiological foundation for improving sperm quality, and significantly increasing the success rate of ART [3].

In TCM practice, male infertility is classified into eight syndrome types: kidney yin deficiency, kidney yang deficiency, kidney essence deficiency, liver qi stagnation, phlegm‐dampness accumulation, dampness‐heat, qi stagnation with blood stasis and spleen deficiency with dampness exuberance [5, 6]. In clinical cases, pure kidney deficiency is rare, with the kidney deficiency accompanied by dampness‐heavy pattern predominating. Youjing Granules (YGs), a time‐tested formula developed by Professor Xu Fusong, one of the founders of andrology in TCM, is used for treating male infertility. Its composition includes *Astragali radix* (Huangqi, HQ), *Dioscorea spongiosa rhizome* (Bijie, BJ), *Cuscutae semen* (Tusizi, TSZ), *Astragali Complanati Semen* (Shayuanzi, SYZ), *Plantaginis semen* (Cheqianzi, CQZ), *Acori tatarinowii rhizome* (Shichangpu, SCP), *Gleditsiae spina* (Zaojiaoci, ZJC), *Dipsaci radix* (Xuduan, XD), *Ostreae concha* (Muli, ML), *Imperatae rhizoma* (Baimaogen, BMG), *Poria* (Fulin, FL), *Polygoni multiflori radix praeparata* (Zhiheshouwu, ZSW), *Moutan Cortex* (Mudanpi, MDP) and *Stir‐fried Atractylodis Macrocephalae Rhizoma* (Chaobaizhu, CBZ). This formula, based on the combination of tonifying the kidney, resolving dampness, removing blood stasis and clearing heat, has achieved favourable outcomes in clinical application over many years. Its efficacy and mechanisms of action have been verified in previous studies [7]. Preliminary studies indicate that YG upregulates the expression of molecules related to self‐renewal and proliferation in spermatogonia stem cells (SSCs), such as neurotrophic factors and fibroblast growth factor‐2 [8, 9]. This helps ameliorate sperm quality and promote the proliferation and self‐renewal of SSCs, thereby protecting spermatogenic function in rats. YG exhibits unique advantages in the treatment of male infertility. However, the composition of TCM formulas is complex, and their pharmacological effects result from the synergistic actions of multiple active constituents. The specific active ingredients in YG responsible for its efficacy remain unclear.

To establish a dedicated chemical component database for YG and clarify ‘what is in it’, this study employed ultra‐high‐performance liquid chromatography coupled with hybrid quadrupole‐orbitrap high‐resolution mass spectrometry (UHPLC‐Q‐Orbitrap‐MS) technology to comprehensively analyse and identify the chemical components and blood‐absorbed components of YG, aiming to provide a reference basis for research on the pharmacodynamic material basis of YG.

## 2 Materials and Methods

### 2.1 Instruments and Reagents

Vanquish Flex UHPLC ultra‐high‐performance liquid chromatograph, Q Exactive Quadrupole‐Orbitrap Mass Spectrometer (Thermo Fisher Scientific, USA); ACQUITY UPLC HSS T3 reversed‐phase chromatographic column (Waters Corporation, USA); Mikro 220R high‐speed refrigerated centrifuge (Hettich Lab Technology, Germany); KQ3200D Ultrasonic Extractor (Kunshan Ultrasonic Instrument Co. Ltd., China).

*Astragali radix* (No.: 2412109101), *Dioscorea spongiosa rhizome* (No.: 2410113101), *Cuscutae semen* (No.: 2412156101), *Astragali Complanati Semen* (No.: 2412266101), *Plantaginis semen* (No.: 2412135101), *Acori tatarinowii rhizome* (No.: 2502368101), *Gleditsiae spina* (No.: 2412196101), *Dipsaci radix* (No.: 2412159101), *Ostreae concha* (No.: 2502047101), *Imperatae rhizoma* (No.: 2409379101), Poria (No.: 2408169101), *Polygoni multiflori radix praeparata* (No.: 2412045101), *Moutan Cortex* (No.: 2503560101), *Stir‐fried Atractylodis Macrocephalae Rhizoma* (No.: 2504252101). All granules were provided by Tianjiang Pharmaceutical Co. Ltd. Methanol, acetonitrile and acetic acid were all mass spectrometry grade (Thermo Fisher Scientific, USA).

### 2.2 Experimental Animals

Twelve SPF‐grade male SD rats weighing 220–250 g were purchased from Guangzhou RuiGe Biotechnology Co. Ltd. Animal Production License Number: SCXK (Yue) 2023‐0059. Housing conditions: Temperature 23–26°C, relative humidity 45%–55%, with a 12‐h light/dark cycle. All animal procedures were approved by the Ethics Committee of Yangzhou University (No.: 2017‐065).

### 2.3 Preparation of Drug Solution

An appropriate amount of YG was placed in a glass test tube, dissolved with 10 times the amount of hot purified water under stirring and prepared into a 0.2‐g/mL YG solution for gavage administration to rats. A volume of 100 µL of YG sample and 300 µL methanol were charged into a 1.5‐mL centrifuge tube and vortexed for 1 min. After that, the tube was centrifuged at 4°C and 12,000 rpm for 10 min. Finally, 100 µL of supernatant was mixed with 100 ultrapure water and then pipetted into an injection vial for detection.

### 2.4 Preparation and Processing of Serum Samples

SD rats were randomly divided into two groups: YG group (*n* = 6) and blank group (*n* = 6). The YG group received YG solution (0.2 g/mL) via gavage administration at a dose of 10 mL/kg, whereas the blank group received an equivalent volume of distilled water. Gavage was administered twice daily for three consecutive days. Blood samples were collected from the abdominal aorta 1 h after the last administration.

A volume of 100 µL of serum and 300 µL of methanol were transferred into a 1.5‐mL centrifuge tube and vortexed for 10 min. The tube was then centrifuged at 4°C and 12,000 rpm for 10 min. A volume of 270 µL of supernatant was charged into a 1.5‐mL centrifuge tube and concentrated by vacuum centrifuge for 4 h. Subsequently, 90 µL 50% methanol water solution was added into the tube and vortexed for 1 min. The tube was then centrifuged at 4°C and 12,000 rpm for 10 min. A volume of 80 µL of supernatant was transferred into an injection vial for analysis.

### 2.5 Chromatographic Methods

A Vanquish Flex UHPLC chromatograph (Thermo Fisher Scientific, Waltham, MA, USA) equipped with an ACQUITY UPLC HSS T3 column (2.1 mm × 100 mm, 1.7 µm) (Waters Corp., Milford, MA, USA) was used for separation. The mobile phase was consisted of water (0.1% formic acid, phase A) and acetonitrile (phase B) with a flow rate of 0.3 mL/min, and the column temperature was 40°C. Elution gradient: 0–1.0 min, 98% A; 1.0–14.0 min, 98% A→70% A; 14.0–25.0 min, 70% A → 0% A; 25.0–28.0 min, 0% A; 28.1–30.0 min, 0% A → 98% A. The injection volume was 6.0 µL.

### 2.6 Mass Spectrometry Methods

The MS data were collected by a hybrid quadrupole orbitrap mass spectrometer (Q Exactive, Thermo Fisher Scientific, Waltham, MA, USA) equipped with a HESI‐II spray probe. The parameters were set as follows: positive ion source voltage 3.7 kV and negative ion source voltage 3.5 kV, heated capillary temperature 320°C, sheath gas pressure 30 psi, auxiliary gas pressure 10 psi and desolvation temperature 300°C. Both the sheath gas and the auxiliary gas were nitrogen. The collision gas was also nitrogen with a pressure of 1.5 mTorr. The data were acquired in ‘Full scan/dd‐MS2’ mode. The parameters of the full scan were set as follows: resolution 70,000 auto gain control target 1 × 106, maximum isolation time 50 ms and *m/z* scan range 100–1500. The dd‐MS2 data were collected with the parameters of resolution 17,500 auto gain control target 1 × 105, maximum isolation time 50 ms, top *n* (*n* ≤ 10) most intense parent ions selected for fragmentation coupled with dynamic exclusion mechanism, isolation window of *m/z* 2, collision energy 10, 30 and 60 V and intensity threshold 1 × 105.

### 2.7 Precision

The same YG sample solution was taken and injected six consecutive times according to the experimental analytical method. The relative standard deviation (RSD) of the peak areas of the main components was calculated, and all results were less than 3.0%, indicating that the instrument precision of the analytical method was satisfactory and met the analytical requirements.

### 2.8 Stability

The same YG sample solution was stored at 25°C and analysed at 0, 2, 4, 8, 12 and 24 h, respectively. The variation in peak area of the main components was used for calculation. The results showed that the RSD of the peak areas for all target components within 24 h was less than 5.0%, demonstrating that the test sample solution remained chemically stable at room temperature for 24 h and met the requirements of the analytical process.

### 2.9 Data Processing and Analysis

The MS data were processed by Progenesis QI 3.0 (Waters Corp., Milford, MA, USA) with the steps of raw data introduction, peak extraction and deconvolution. The identification was finally determined by consideration of retention time error of reference substance, mass error of parent ion, match degree of daughter ions, isotope distribution and peak area after searching the reference substance database (TCM Pro 2.0, Beijing Hexin Technology Co. Ltd.) and theoretical database constructed by literature and public databases.

## 3 Results

### 3.1 Chemical Component Analysis of YG

**FIGURE 1:**
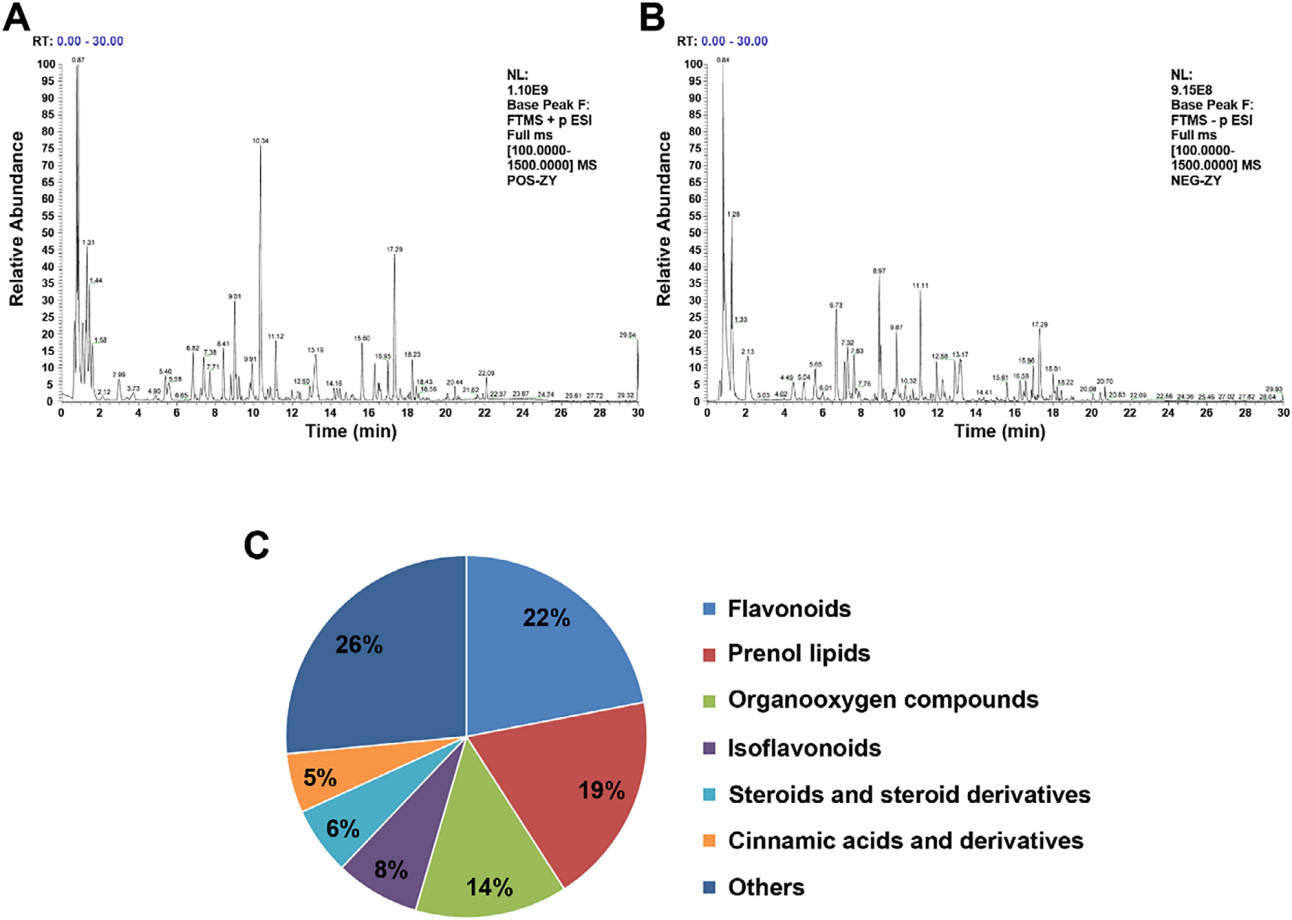
Base peak ions (BPI) chromatograms: (A) YG sample in positive mode; (B) YG sample in negative mode; and (C) relative content distribution of chemical component categories in YG.

UHPLC‐Q‐orbitrap‐MS was conducted for the comprehensive chemical component analysis of YG samples. The base peak ion (BPI) chromatograms of samples in positive and negative ion modes are shown in Figure 1A,B. The major chemical components achieved good separation within 30 min. On the basis of retention time and fragmentation patterns of detected components, comparison with reference standards, relevant literature and databases led to the identification of 132 chemical components. These include 29 flavonoids, 25 prenol lipids, 18 organic oxygen compounds, 10 isoflavonoids, 8 steroids and steroid derivatives, 7 cinnamic acids and derivatives and 35 other compounds. The identification results are presented in Table 1, with the relative content distribution of chemical classifications illustrated in Figure 1C.

**TABLE 1:** Chemical composition of Youjing Granule (YG).

No.	Model	*t*_R_ (min)	Adducts	Experimental *m/z*	Theoretical *m*/*z*	Error (ppm)	Fragments	Formula	Identification	Source	PubChem
1	POS	0.78	[M + H]+	180.0869	180.0866	1.21	180.0872, 162.0760, 96.0453, 87.0451, 85.0295	C6H13NO5	d‐Glucosamine	XD, SYZ, CBZ, SCP, BX, HQ	2723635
2	POS	0.80	[M + H]+	133.0609	133.0608	1.27	74.0249, 87.0563, 88.0403, 116.0348	C4H8N2O3	l‐Asparagine	CBZ, BMG, ZSW	6267
3	POS	0.87	[M + H‐H2O]+	487.1660	487.1657	0.45	325.1130, 163.0601, 145.0496, 127.0393, 97.0292, 85.0295, 69.0348	C18H32O16	Maltotriose	CBZ, MDP, CQZ	439586
4	NEG	0.91	[M + FA‐H]−	387.1142	387.1144	−0.57	341.1085, 179.0544, 161.0435, 119.0326, 113.0219, 101.0218, 96.9575	C12H22O11	Sucrose	CBZ, XD, SYZ, CQZ, HQ, MDP	5988
5	POS	1.31	[M + NH4]+	394.1712	394.1708	1.15	179.0703, 151.0755, 133.0650, 394.1740, 105.0704	C16H24O10	8‐Debenzoylpaeoniflorin	MDP	71452333
6	NEG	1.37	[M − H]−	243.0616	243.0623	−2.59	243.0618, 200.0550, 152.0332, 140.0330, 110.0222, 82.0272, 66.0322	C9H12N2O6	Uridine	SCP, FL, CBZ, HQ	6029
7	NEG	1.46	[M + FA‐H]−	312.0951	312.0950	0.37	134.0448, 266.0896, 107.0338, 59.0112	C10H13N5O4	Adenosine	SCP, FL, BMG, BX, CQZ	60961
8	POS	1.50	[M + H]+	252.1091	252.1091	−0.20	136.0618, 117.0551, 252.1080, 144.9611	C10H13N5O3	Cordycepin	SCP	6303
9	NEG	1.55	[M − H]−	282.0843	282.0844	−0.36	150.0399, 282.0841, 133.0132, 108.0180	C10H13N5O5	Guanosine	SCP, FL, SYZ, CBZ, HQ	135398635
10	POS	1.97	[M + H]+	171.0289	171.0288	0.51	171.0287, 170.0957, 170.0832, 154.0498, 153.0184, 149.9403, 148.9771	C7H6O5	Gallic acid	ZSW, MDP, CQZ, SCP	370
11	NEG	5.04	[M − H]−	373.1139	373.1140	−0.36	373.1144, 211.0600, 167.0694, 149.0586, 123.0428, 89.0216, 71.0112	C16H22O10	Geniposidic acid	CQZ	443354
12	POS	5.40	[M + H]+	205.0972	205.0972	0.16	188.0708, 146.0602, 149.0235, 118.0657, 144.0810	C11H12N2O2	l‐Tryptophan	BX	6305
13	NEG	5.65	[M − H]−	353.0878	353.0878	−0.08	191.0548, 135.0428, 179.0334, 353.0868	C16H18O9	Neochlorogenic acid	XD, CBZ, BMG, TSZ, BX	5280633
14	POS	6.00	[M + H]+	139.0391	139.0390	0.59	139.0391, 137.0599, 116.9668, 111.0447, 95.0864, 93.0345, 81.0710	C7H6O3	Protocatechualdehyde	FL, SCP	8768
15	POS	6.56	[M + H]+	579.1501	579.1497	0.74	579.1503, 427.1019, 291.0856, 289.0692, 287.0543, 271.0608, 247.0608	C30H26O12	Procyanidin B1	MDP, BX	11250133
16	POS	6.80	[M + H]+	247.1439	247.1441	−0.81	188.0707, 146.0601, 60.0822, 118.0656, 144.0811	C14H18N2O2	Hypaphorine	—	442106
17	POS	6.82	[M + NH4]+	394.1706	394.1708	−0.33	215.0915, 197.0809, 179.0703, 151.0756, 137.0598, 133.0650, 123.0809	C16H24O10	Loganic acid	XD	89640
18	POS	7.22	[2M + H]+	753.2810	753.2812	−0.26	377.1442, 215.0915, 197.0810, 179.0704, 153.0547, 151.0754, 151.0392	C16H24O10	8‐Epi‐loganic acid	CQZ	158144
19	POS	7.38	[M + H‐H2O]+	163.0388	163.0390	−0.76	163.0390, 135.0442, 145.0286, 117.0340, 89.0396	C9H8O4	Caffeic acid	BMG, XD, SYZ, CBZ, BX, MDP, ZJC, SCP, TSZ, HQ	689043
20	NEG	7.38	[M − H]−	495.1507	495.1508	−0.27	137.0221, 495.1513, 93.0320, 165.0534	C23H28O12	Oxypaeoniflorin	MDP, FL	21631105
21	NEG	7.63	[M − H]−	353.0877	353.0878	−0.24	353.0878, 191.0546, 179.0333, 173.0437, 135.0429, 93.0320, 85.0269	C16H18O9	Cryptochlorogenic acid	XD, CBZ, BMG, TSZ, SYZ, BX, CQZ	9798666
22	POS	7.71	[M + H]+	355.1022	355.1024	−0.50	163.0390, 135.0442, 145.0285, 91.0582	C16H18O9	Chlorogenic acid	XD, CBZ, BMG, TSZ, BX, HQ, SCP	1794427
23	NEG	7.79	[M − H]−	367.1033	367.1035	−0.43	193.0491, 134.0350, 367.1032, 117.0325	C17H20O9	5‐*O*‐Feruloylquinic acid	BMG	10133609
24	POS	8.99	[M + H]+	359.1332	359.1337	−1.37	197.0809, 127.0393, 179.0702, 111.0810	C16H22O9	Sweroside	XD, MDP, BX	161036
25	POS	9.08	[M + H]+	229.1069	229.1070	−0.66	197.0809, 179.0703, 161.0597, 151.0755, 137.0599, 133.0650, 123.0808	C11H16O5	Loganetin	XD	10466307
27	POS	9.08	[M + NH4]+	408.1862	408.1864	−0.50	179.0703, 229.1070, 109.0654, 151.0755, 81.0710, 133.0649	C17H26O10	Loganin	XD	87691
28	POS	9.10	[M + H]+	595.1659	595.1657	0.28	595.1658, 577.1542, 457.1158, 439.1045, 427.1031, 421.0912, 409.0915	C27H30O15	Vicenin 2	SYZ, BX, CQZ, HQ	442664
29	NEG	9.17	[M + FA‐H]−	505.1564	505.1563	0.34	165.0537, 150.0301, 459.1512, 293.0879	C20H28O12	Paeonolide	MDP	442923
30	NEG	9.19	[M + FA‐H]−	373.1139	373.1140	−0.23	165.0538, 150.0303, 373.1129, 160.8399	C15H20O8	Paeonoside	MDP	442924
31	NEG	9.34	[M + FA‐H]−	525.1615	525.1614	0.34	121.0271, 525.1586, 479.1560, 167.0335	C23H28O11	Albiflorin	MDP, FL	24868421
32	POS	9.35	[M + H]+	613.1401	613.1399	0.32	319.0449, 153.0183, 481.0981, 85.0295	C26H28O17	Myricetin 3‐*O*‐β‐d‐xylopyranosyl(1‐2)‐β‐d‐glucopyranoside	SYZ	101641678
33	POS	9.53	[M + H]+	153.0546	153.0546	−0.01	153.0548, 131.9745, 125.0601, 121.0289, 113.9643, 111.0447, 109.1019	C8H8O3	Isovanilline	SYZ, CBZ, BMG, FL, BX	12127
34	POS	9.69	[M + H]+	369.1179	369.1180	−0.17	177.0547, 145.0285, 117.0340, 91.0586	C17H20O9	4‐*O*‐Feruloylquinic acid	CBZ, BMG, BX	10177048
35	NEG	9.69	[M − H]−	405.1190	405.1191	−0.21	243.0656, 405.1191, 137.0222, 173.0589	C20H22O9	Piceatannol 3′‐*O*‐glucoside	ZSW	11968990
36	POS	9.71	[M + H]+	165.0546	165.0546	0.13	147.0442, 119.0497, 91.0552, 163.0390	C9H8O3	*p*‐Coumaric acid	BMG, BX, SCP, HQ, CQZ	637542
37	POS	9.76	[M + H]+	565.1554	565.1552	0.35	565.1547, 547.1442, 409.0911, 391.0818, 379.0819, 361.0703, 349.0702	C26H28O14	Vicenin I	SYZ, CBZ, BMG, SCP, HQ	13644663
38	NEG	9.87	[M + FA‐H]−	525.1610	525.1614	−0.85	121.0270, 449.1458, 525.1639, 479.1563	C23H28O11	Paeoniflorin	MDP, FL	442534
39	POS	9.96	[M + H]+	417.1178	417.1180	−0.40	255.0651, 199.0755, 417.1181, 227.0702	C21H20O9	Daidzin	SYZ	107971
40	NEG	9.99	[M − H]−	563.1405	563.1406	−0.19	563.1403, 353.0668, 443.0985, 297.0771, 383.0768, 473.1097	C26H28O14	Schaftoside	SYZ, CBZ, CQZ, SCP	442658
41	POS	10.09	[M + H]+	449.1077	449.1078	−0.21	449.1085, 431.0977, 413.0864, 395.0764, 365.0661, 353.0658, 339.0862	C21H20O11	Isoorientin	CQZ	114776
133	NEG	10.10	[M − H]^−^	287.0561	287.0561	−0.09	287.0556, 269.0462, 259.0613, 225.0544, 165.0172, 163.0016, 153.0169	C_15_H_12_O_6_	2,3‐Dihydrofisetin	SYZ, ZJC	5317435
42	NEG	10.22	[M − H]−	447.0935	447.0933	0.45	447.0932, 357.0613, 327.0510, 299.0553, 298.0474, 297.0405, 133.0270	C21H20O11	Orientin	—	5281675
43	POS	10.40	[M + H]+	183.0652	183.0652	−0.10	183.0652, 159.9692, 155.0703, 140.0468, 131.9744, 123.0444, 113.9643	C9H10O4	3,5‐Dimethoxy‐4‐hydroxybenzaldehyde	SYZ	8655
44	POS	10.58	[M + H]+	465.1028	465.1028	0.13	303.0498, 61.0299, 85.0295, 229.0495	C21H20O12	Hyperoside	TSZ, ZSW, HQ	5281643
45	POS	10.61	[M + NH4]+	760.3017	760.3022	−0.72	419.1691, 401.1588, 265.1068, 235.0962, 217.0860, 205.0861, 190.0624	C34H46O18	Eleutheroside E	XD, SYZ	71312557
46	NEG	10.65	[M − H]−	611.1620	611.1618	0.41	611.1613, 445.0984, 343.0666, 301.0557, 193.0129, 169.0121, 165.0537	C27H32O16	Suffruticoside C	MDP	10258206
47	POS	10.79	[M + H]+	193.0496	193.0495	0.20	193.0498, 133.0286, 178.0261, 137.0598	C10H8O4	Isoscopoletin	CBZ	69894
48	NEG	10.80	[M − H]−	555.1720	555.1719	0.20	151.0379, 479.1558, 555.1722, 165.0538	C25H32O14	10‐Hydroxyoleuropein	SYZ	6440747
49	POS	10.83	[M + H‐H2O]+	177.0546	177.0546	−0.23	145.0285, 117.0340, 177.0547, 89.0396, 149.0599	C10H10O4	Ferulic acid	BMG, SYZ, SCP, TSZ, HQ, ZSW, CQZ	445858
50	NEG	11.04	[M − H]−	431.0981	431.0984	−0.55	311.0562, 431.0983, 283.0610, 161.0224, 341.0686	C21H20O10	Vitexin	SYZ	5280441
51	NEG	11.07	[M − H]−	577.1565	577.1563	0.34	293.0454, 577.1576, 413.0911, 59.0112	C27H30O14	Vitexin rhamnoside	—	5282151
52	NEG	11.08	[M + FA‐H]−	491.1192	491.1195	−0.67	283.0612, 268.0377, 491.1203, 240.0426	C22H22O10	Calycosin‐7‐*O*‐β‐d‐glucoside	HQ, SYZ	5318267
53	POS	11.10	[M + H]+	609.1812	609.1814	−0.31	609.1808, 351.0851, 327.0860, 323.0914, 308.0667, 297.0757, 285.0751	C28H32O15	Spinosin	BX, XD	155692
54	POS	11.12	[M + H]+	407.1333	407.1337	−0.99	245.0808, 199.0755, 125.0237, 151.0390, 227.0702, 85.0295	C20H22O9	2,3,5,4′‐Tetrahydroxy stilbene‐2‐*Ο*‐β‐d‐glucoside	ZSW	5321884
55	POS	11.16	[M + H]+	305.0651	305.0656	−1.59	304.0533, 287.0550, 259.0604, 231.0654, 195.0292, 153.0183, 149.0236	C15H12O7	Taxifolin	ZJC, BX, HQ	439533
56	POS	11.19	[M + H]+	433.1129	433.1129	−0.09	433.1135, 415.1028, 379.0813, 367.0808, 349.0713, 337.0705, 323.0922	C21H20O10	Isovitexin	SYZ, ZJC	162350
57	POS	11.28	[M + H]+	581.1501	581.1501	0.01	287.0549, 449.1082, 85.0295, 73.0297	C26H28O15	Leucoside	SYZ	44566720
58	POS	11.37	[M + H]+	465.1028	465.1028	0.11	303.0497, 85.0295, 229.0496, 153.0183	C21H20O12	Isoquercitrin	TSZ, BX, CQZ, MDP	5280804
59	NEG	11.63	[M − H]−	623.1985	623.1981	0.51	161.0224, 623.1987, 133.0272, 113.0219, 135.0428, 461.1669	C29H36O15	Acteoside	CQZ, HQ	5281800
60	NEG	11.68	[M + FA‐H]−	509.1298	509.1301	−0.52	509.1295, 341.0888, 327.0731, 197.0446, 183.0281, 181.0494, 167.0331	C22H24O11	7‐*O*‐Methyl luteolin‐6‐C‐β‐d‐glucoside	SYZ	5319708
61	POS	11.68	[M + H]+	433.1130	433.1129	0.08	271.0599, 433.1132, 153.0183, 215.0703	C21H20O10	Sophoricoside	SYZ, CQZ, HQ	5321398
62	POS	11.79	[M + NH4]+	658.2345	658.2342	0.46	163.0389, 325.0916, 135.0442, 145.0287	C29H36O16	Plantainoside D	CQZ	9986606
63	POS	11.91	[M + H]+	265.0969	265.0972	−0.80	265.0971, 206.0840, 105.0343, 247.0864	C16H12N2O2	Perlolyrine	XD, BMG	160179
64	POS	11.95	[M + H]+	517.1341	517.1341	0.08	163.0390, 135.0442, 145.0285, 499.1241	C25H24O12	Isochlorogenic acid B	XD, CBZ, BMG, SCP, CQZ, TSZ	5281780
65	NEG	11.99	[M − H]−	447.0933	447.0933	0.08	447.0932, 284.0327, 255.0298, 227.0341, 285.0393	C21H20O11	Astragalin	SYZ, SCP, TSZ	5282102
66	POS	12.04	[M + H]+	595.1658	595.1657	0.09	287.0548, 71.0505, 85.0296, 449.1087	C27H30O15	Nicotiflorin	CQZ, SCP	5318767
67	NEG	12.24	[M − H]−	515.1192	515.1195	−0.66	191.0546, 179.0332, 135.0429, 353.0878, 515.1195	C25H24O12	Isochlorogenic acid A	XD, SYZ, CBZ, BMG, BX, HQ, TSZ	6474310
68	POS	12.29	[M + NH4]+	642.2390	642.2392	−0.38	163.0390, 325.0916, 135.0442, 145.0285	C29H36O15	Isoacteoside	CQZ	6476333
69	POS	12.38	[M + NH4]+	538.2284	538.2283	0.17	341.1384, 235.0965, 205.0855, 187.0754, 175.0755, 160.0520, 137.0598	C26H32O11	(+)‐Pinoresinol 4‐*O*‐glucoside	XD, CQZ, HQ, TSZ	486614
70	NEG	12.67	[M − H]−	577.1566	577.1563	0.57	269.0454, 577.1578, 268.0376, 269.0760	C27H30O14	Isorhoifolin	CQZ	9851181
71	POS	12.67	[M + H]+	579.1708	579.1708	−0.12	271.0599, 579.1703, 153.0181, 119.0496	C27H30O14	Rhoifolin	CQZ, SCP	5282150
72	POS	12.74	[M + H]+	479.1184	479.1184	0.08	317.0656, 302.0423, 85.0295, 153.0179	C22H22O12	Isorhamnetin‐3‐*O*‐glucoside	—	5318645
73	POS	12.86	[M + NH4]+	540.2074	540.2076	−0.27	168.0655, 540.2076, 193.0860, 161.0597	C25H30O12	6‐p‐Methoxycinnamoyl catalpol	SYZ	6325621
74	NEG	12.88	[M − H]−	515.1191	515.1195	−0.81	515.1191, 353.0878, 191.0546, 179.0333, 173.0437, 135.0428, 93.0319	C25H24O12	Isochlorogenic acid C	XD, SYZ, CBZ, SCP, BX, HQ, TSZ	6474309
75	NEG	12.88	[M + FA‐H]−	801.2102	801.2095	0.88	283.0246, 298.0481, 593.1516, 755.2043, 255.0293	C33H40O20	Complanatoside B	SYZ	10440090
76	NEG	13.17	[M + FA‐H]−	791.2621	791.2615	0.76	745.2580, 583.2037, 513.1604, 459.1519, 209.0804, 193.0483, 141.0172	C33H46O19	Cantleyoside	XD	12302406
77	POS	13.30	[M + H]+	463.1231	463.1235	−0.83	301.0705, 463.1232, 286.0472, 258.0522	C22H22O11	Diosmetin‐7‐*O*‐β‐d‐glucopyranoside	SYZ, BX, CQZ, HQ	11016019
78	NEG	13.30	[M + FA‐H]−	669.1678	669.1672	0.98	299.0558, 461.1091, 283.0247, 271.0609, 165.0172, 298.0478	C28H32O16	Complanatuside	SYZ	5492406
79	NEG	13.67	[M − H]−	431.0983	431.0984	−0.18	269.0454, 431.0978, 240.0422, 225.0549	C21H20O10	Oroxin A	HQ	5320313
80	NEG	14.01	[M − H]−	717.1470	717.1461	1.31	717.1467, 519.0928, 339.0501, 321.0403, 295.0601, 293.0448, 279.0306	C36H30O16	Salvianolic acid B	—	11629084
81	POS	14.16	[M + H]+	431.1335	431.1337	−0.44	269.0807, 431.1346, 254.0563, 213.0910	C22H22O9	Ononin	SYZ, BMG, HQ	442813
82	POS	14.16	[M + H]+	284.1277	284.1281	−1.35	147.0441, 121.0652, 284.1282, 119.0496	C17H17NO3	*N*‐*p*‐*trans*‐Coumaroyltyramine	—	5372945
83	NEG	14.17	[M − H]−	445.0777	445.0776	0.15	269.0454, 445.0779, 113.0220, 85.0268	C21H18O11	Baicalin	SYZ, SCP, HQ, ZSW, CQZ	64982
84	POS	14.34	[M + H]+	197.0807	197.0808	−0.62	197.0809, 169.0859, 154.0625, 139.0391, 138.0676, 131.9744, 123.0444	C10H12O4	2,4,5‐Trimethoxybenzaldehyde	SCP	20525
85	NEG	14.41	[M − H]−	599.1773	599.1770	0.53	599.1777, 447.1276, 431.1352, 429.1174, 281.0667, 179.0330, 149.0585	C30H32O13	Mudanpioside C	MDP	21631098
86	POS	14.47	[M + H]+	331.1536	331.1540	−1.19	331.1541, 313.1436, 287.1274, 285.1118, 255.1016, 253.1229, 253.0866	C19H22O5	Magnolignan D	CBZ	5319189
87	NEG	14.53	[M − H]−	253.0503	253.0506	−1.27	253.0502, 224.0460, 217.8488, 209.0590	C15H10O4	Daidzein	SYZ, HQ	5281708
88	POS	15.06	[M + NH4]+	618.2178	618.2181	−0.58	317.1013, 267.0854, 249.0757, 179.0706, 151.0757, 123.0442, 105.0343	C30H32O13	Benzoyloxypaeoniflorin	MDP	21631107
89	NEG	15.09	[M + FA‐H]−	1109.5385	1109.5385	−0.08	1063.5331, 101.0218, 1061.5188, 163.0591, 205.0708, 917.4738	C51H84O23	Protogracillin	BX	441892
90	POS	15.13	[M + H‐H2O]+	301.1067	301.1070	−1.01	167.0704, 301.1068, 152.0468, 134.0364	C17H18O6	Paeonilactone‐C	SYZ, MDP	10471123
91	POS	15.42	[M + H]+	303.0495	303.0499	−1.53	303.0497, 123.0445, 167.0704, 153.0180	C15H10O7	Quercetin	ZJC, ZSW, MDP	5280343
92	POS	15.60	[M + H]+	285.0752	285.0757	−1.83	285.0755, 270.0520, 225.0545, 253.0494	C16H12O5	Calycosin	SYZ, HQ	5280448
93	POS	15.69	[M + H]+	243.1013	243.1016	−1.21	243.1015, 107.0498, 135.0442, 134.0728, 211.0763, 121.0651	C15H14O3	4′‐Methoxyresveratrol	非药味特征化合物/复方	6255462
94	NEG	15.71	[M − H]−	329.0303	329.0303	0.14	329.0309, 314.0066, 270.9882, 298.9836, 312.9996	C16H10O8	3,4′‐Di‐*O*‐methylellagic acid	SYZ	5491816
95	NEG	15.98	[M − H]−	431.0981	431.0984	−0.67	269.0454, 431.0984, 225.0545, 240.0418	C21H20O10	Emodin‐8‐β‐d‐glucoside	XD, HQ	99649
96	NEG	16.28	[M + FA‐H]−	461.1088	461.1089	−0.25	461.1090, 299.0554, 298.0480, 283.0603, 283.0251, 268.0376, 255.0295	C21H20O9	Chrysophanein	SYZ	6324923
97	POS	16.28	[M + H‐H2O]+	1031.5404	1031.5421	−1.61	1031.5417, 85.0294, 253.1948, 129.0548	C51H84O22	Protodioscin	BX	441891
98	POS	16.86	[M + H]+	271.0597	271.0601	−1.65	271.0601, 153.0182, 215.0691, 243.0657	C15H10O5	Genistein	SYZ	5280961
99	NEG	16.88	[M + FA‐H]−	1281.6113	1281.6121	−0.67	911.5015, 1235.6063, 603.3905, 471.3477, 749.4485	C59H96O27	Macranthoidin A	XD	14564503
100	POS	16.95	[M + NH4]+	602.2228	602.2232	−0.68	105.0342, 179.0702, 267.0862, 151.0755, 249.0756, 81.0346	C30H32O12	Benzoylpaeoniflorin	MDP, FL	21631106
101	NEG	17.06	[M − H]−	971.4869	971.4857	1.23	603.3904, 927.4962, 601.3751, 971.4858	C48H76O20	Sophoraflavoside II	SYZ	197561
102	NEG	17.12	[M + FA‐H]−	1119.5597	1119.5593	0.37	749.4485, 1073.5543, 1119.5608, 471.3478	C53H86O22	Dipsacoside B	XD	21627940
103	NEG	17.29	[M + FA‐H]−	973.5017	973.5014	0.40	603.3907, 973.5012, 927.4973, 323.0985	C47H76O18	Asperosaponin VI	XD	14284436
104	NEG	17.50	[M − H]−	299.0559	299.0561	−0.83	299.0560, 284.0323, 285.0374, 148.0144	C16H12O6	4′‐Hydroxywogonin	SYZ	5322078
105	POS	17.60	[M + H]+	167.0701	167.0703	−0.91	167.0704, 149.0597, 121.0654, 125.0607	C9H10O3	Paeonol	MDP, BMG, BX, SCP	11092
106	POS	17.69	[M + NH4]+	1048.5681	1048.5687	−0.53	1048.5692, 1031.5424, 869.4933, 723.4330, 577.3739, 415.3207, 271.2054	C51H82O21	Pseudoprotodioscin	BX	51346147
107	NEG	17.73	[M − H]−	971.4868	971.4857	1.06	601.3748, 603.3902, 925.4823, 971.4868	C48H76O20	Dianoside G	SYZ	125937
108	NEG	18.01	[M − H]−	329.2331	329.2333	−0.72	329.2334, 211.1329, 229.1437, 171.1008	C18H34O5	9,10,13‐Trihydroxy‐11‐octadecenoic acid	SYZ, CBZ, FL	5282965
109	NEG	18.01	[M + FA‐H]−	991.5126	991.5119	0.74	991.5121, 945.5077, 119.0325, 113.0217, 101.0218, 89.0218, 71.0111	C47H78O19	Astragaloside V	HQ	71448939
110	NEG	18.22	[M − H]−	267.0660	267.0663	−0.89	267.0661, 252.0423, 223.0391, 251.0344	C16H12O4	Formononetin	SYZ, HQ	5280378
111	POS	18.43	[M + NH4]+	930.5406	930.5421	−1.59	930.5421, 439.3575, 205.1953, 203.1796, 191.1794, 189.1638, 145.0496	C47H76O17	Soyasaponin II	SYZ, HQ	443614
112	POS	18.43	[M + H]+	785.4671	785.4682	−1.36	784.5805, 772.2932, 734.2094, 704.4370, 473.3618, 455.3503, 437.3419	C41H68O14	Astragaloside IV	HQ	13943299
113	POS	18.50	[M + H]+	301.1066	301.1070	−1.53	167.0704, 301.1071, 299.0914, 152.0469	C17H16O5	3‐Hydroxy‐9,10‐dimethoxyptercarpan	HQ	14077830
114	POS	18.68	[M + H]+	303.1222	303.1227	−1.67	123.0445, 167.0704, 133.0650, 161.0598	C17H18O5	7,2′‐Dihydroxy‐3′,4′‐dimethoxyisoflavan	HQ	602152
115	NEG	18.94	[M − H]−	1351.6523	1351.6540	−1.24	1351.6537, 1205.5969, 471.3477, 749.4493	C64H104O30	Asperosaponin F	XD	11968864
116	POS	18.97	[M + H]+	943.5252	943.5261	−0.99	943.5297, 943.3523, 894.6891, 865.4182, 797.4710, 781.4672, 635.4149	C48H78O18	Soyasaponin Bb	SYZ, HQ	122097
117	POS	19.17	[M + H]+	301.0703	301.0707	−1.25	283.0600, 199.0752, 301.0711, 227.0703	C16H12O6	Rhamnocitrin	SYZ, HQ	5320946
118	POS	19.59	[M + H]+	209.1171	209.1172	−0.38	209.1172, 207.1744, 194.0939, 189.1639, 181.0860, 179.0711, 178.0994	C12H16O3	β‐Asarone	SCP	5281758
119	POS	20.00	[M + H–H2O]+	231.1377	231.1380	−0.86	231.1380, 213.1270, 189.0911, 185.1326, 175.0755, 163.0754, 161.0597	C15H20O3	Atractylenolide III	CBZ, SYZ, FL	155948
120	NEG	20.00	[M + FA‐H]−	795.4544	795.4536	1.07	795.4571, 749.4501, 603.3874, 471.3483, 89.0217, 71.0111, 59.0113	C41H66O12	α‐Hederin	XD	73296
121	POS	20.04	[M + H]+	417.1904	417.1908	−0.86	417.1893, 249.1125, 219.1027, 208.1097, 193.0861, 181.0859, 165.0547	C23H28O7	Gomisin O	SYZ	5317808
122	POS	20.44	[M + H–H2O]+	437.3412	437.3414	−0.40	437.3423, 215.1796, 203.1798, 201.1641, 189.1639, 159.1172, 147.1170	C30H48O4	Subprogenin A	FL	101605318
123	POS	20.44	[2M + H]+	1209.8022	1209.8023	−0.09	737.4458, 605.4058, 587.3951, 455.3520, 437.3416, 409.3463, 391.3356	C35H56O8	Hederagenin 3‐*O*‐arabinoside	XD	441928
124	POS	20.53	[M + H]+	445.2118	445.2122	−0.78	105.0343, 194.1178, 117.0704, 224.1069, 91.0546, 134.0965	C27H28N2O4	Asperglaucide	XD, CBZ, FL, CQZ	10026486
125	NEG	20.70	[M − H]−	269.0453	269.0455	−0.87	269.0454, 225.0547, 62.9614, 241.0504	C15H10O5	Emodin	ZSW, XD, CBZ, SCP	3220
126	NEG	21.17	[M + FA‐H]−	913.4815	913.4802	1.49	913.4794, 867.4757, 721.4150, 163.0591, 119.0322, 113.0220, 101.0217	C45H72O16	Dioscin	BX, CQZ	119245
127	POS	21.47	[M + H]+	219.1742	219.1743	−0.60	219.1745, 201.1639, 175.1483, 161.1326, 159.1166, 155.9335, 153.9370	C15H22O	Nootkanone	XD, BMG	1268142
128	NEG	21.54	[M + FA‐H]−	315.2538	315.2541	−1.09	315.2537, 116.9259, 297.2439, 141.1258	C17H34O2	Methyl Palmitate	SYZ, CBZ, SCP, FL, CQZ, HQ, ZSW, MDP	8181
129	POS	21.56	[M + H]+	233.1534	233.1536	−0.73	233.1539, 215.1432, 187.1484, 177.0905, 159.0803, 151.0753, 131.0857	C15H20O2	Atractylenolide II	SYZ, CBZ, BMG, FL, BX	14448070
130	NEG	21.69	[M + FA‐H]−	767.4232	767.4223	1.24	767.4221, 721.4141, 264.6903, 251.0151, 249.0591, 215.9853, 166.6230	C39H62O12	Paris saponin V	BX	11061578
131	POS	22.95	[M + H]+	285.0755	285.0757	−0.90	285.0754, 284.3310, 284.2948, 261.8956	C16H12O5	Physcion	ZSW, TSZ	10639
132	POS	23.51	[M + H–H2O]+	467.3521	467.3520	0.27	467.3543, 449.3389, 423.1747, 311.2357, 293.2268, 196.4135, 140.6940	C31H48O4	Dehydrotumulosic acid	FL	15225964

### 3.2 Fragmentation Patterns of Compounds

#### 3.2.1 Flavonoids

A total of 29 flavonoids were identified in the YG sample, 23 of which belonged to flavonoid glycosides. In negative ion mode, flavonoids readily form [M − H]^−^ quasi‐molecular ions. During fragmentation, glycosidic bond cleavage frequently occurs, losing glucose or rhamnose molecules to yield aglycone fragment ions such as [M − H–Glc]^−^ or [M − H–Rha]^−^. These aglycone fragments subsequently undergo further neutral molecule losses (e.g., CH_3_, CO, H_2_O and CHO).

**FIGURE 2:**
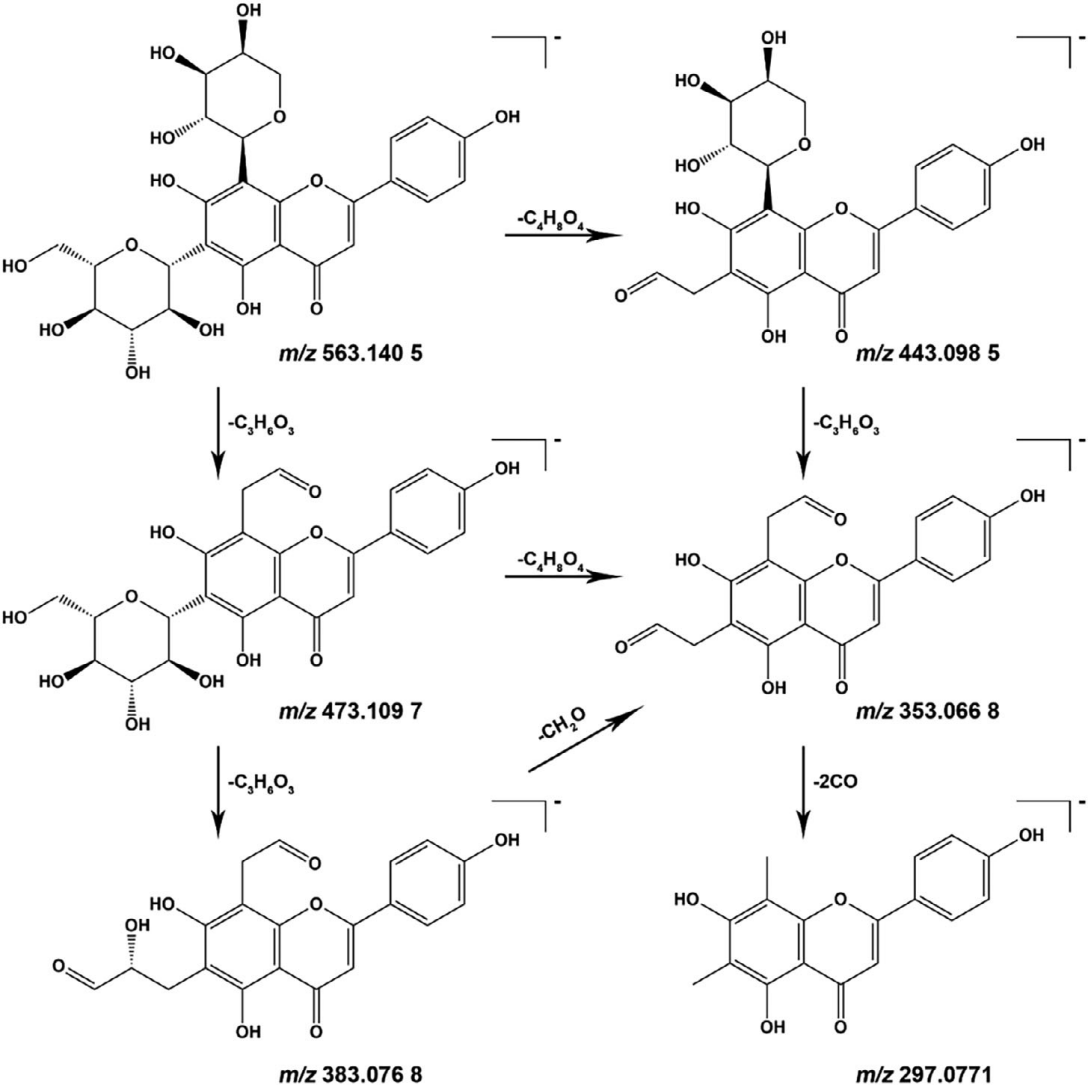
Fragmentation pathway of schaftoside.

Compound 39 serves as an illustrative example. In negative ion mode, the quasi‐molecular ion of this compound was observed at *m/z* 563.1405 [M − H]^−^, and the molecular formula was determined as C_26_H_28_O_14_ through mass spectrometry software fitting. On the basis of fragment ion *m/z* values at 473.1097, 443.0985, 383.0768, 353.0668 and 297.0771, we deduced the formation of fragment ions such as [M − H–C_4_H_8_O_4_]^−^, [M − H–C_4_H_8_O_4_–C_3_H_6_O_3_]^−^ or [M − H–C_3_H_6_O_3_–C_4_H_8_O_4_]^−^, [M − H–C_3_H_6_O_3_]^−^, [M − H–2C_3_H_6_O_3_]^−^ and [M − H–C_4_H_8_O_4_–C_3_H_6_O_3_–2CO]^−^ or [M − H–C_3_H_6_O_3_–C_4_H_8_O_4_–2CO]^−^. This analysis was further supported by database matching and literature reports [10, 11]. This compound was tentatively identified as schaftoside, and its fragmentation pathway is shown in Figure 2.

#### 3.2.2 Prenol Lipids

A total of 25 prenol lipids compounds were detected in YG samples, of which 20 belonged to terpene glycosides. In negative ion mode, these compounds can readily form quasi‐molecular ions such as [M − H]^−^ and [M + HCOO]^−^, which can easily remove the glucose structure during fragmentation, generating glycoside fragment ions [M − H–Glc]^−^. Subsequently, the glycoside fragments further lose H_2_O and CO_2_ molecules, generating fragment ions such as [M − H–Glc–H_2_O]^−^, [M − H–Glc–CO_2_]^−^ and [M − H–Glc–H_2_O–CO_2_]^−^.

**FIGURE 3:**
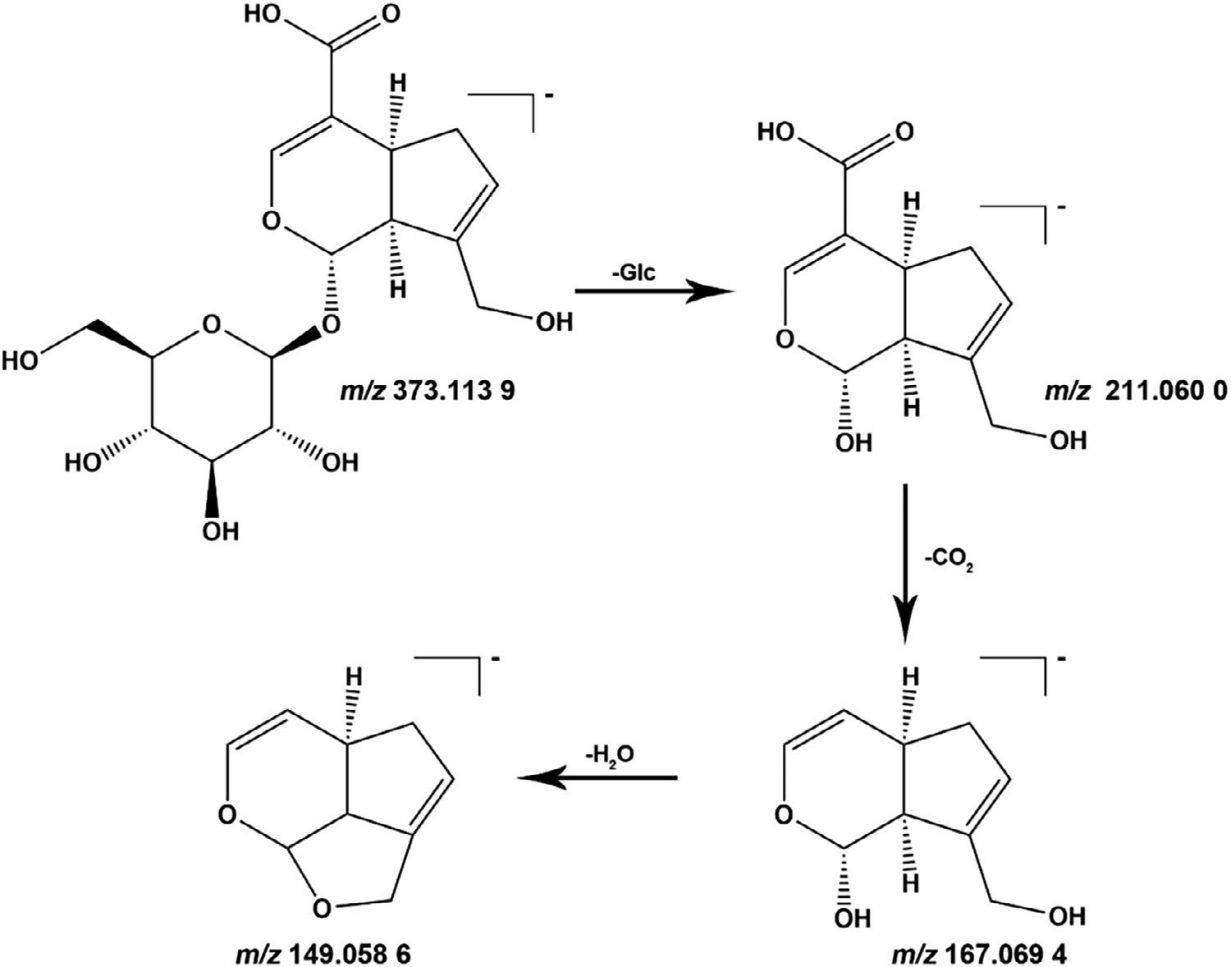
Fragmentation pathways of geniposidic acid.

Taking Compound 11 as an example, the quasi‐molecular ion of this compound was observed at *m/z* 373.1139 [M − H]^−^. The molecular formula was determined as C_16_H_22_O_11_ through accurate mass measurement. On the basis of fragment ions at *m/z* 211.0600, 167.0694 and 149.0586, we inferred the formation of fragment ions, including [M − H–Glc]^−^, [M − H–Glc–CO_2_]^−^ and [M − H–Glc–H_2_O–CO_2_]^−^. This fragmentation pattern suggests the presence of a glucose moiety and an iridoid aglycone structure. Through database cross‐referencing and literature comparison, the compound was ultimately identified as azadirachtin [12, 13, 14]. Its fragmentation pathway is illustrated in Figure 3.

#### 3.2.3 Organooxygen Compounds

A total of 18 organooxygen compounds were detected in YG samples, of which 8 fell into the categories of alcohols and polyols, and another 8 belonged to carbohydrates and their derivatives. In negative ion mode, it readily forms [M − H]^−^ quasi‐molecular ions. Fragment ions of these compounds are primarily generated through cleavage of ester bonds and glycosidic bonds. Cleavage at ester bonds results in the loss of groups such as caffeoyl (C_9_H_6_O_3_) and feruloyl (C_10_H_8_O_3_). Cleavage at the glycosidic bond results in the loss of a glucose residue (C_6_H_10_O_5_). Additionally, the loss of common neutral molecules such as H_2_O, CO_2_ and CO can also be observed.

**FIGURE 4:**
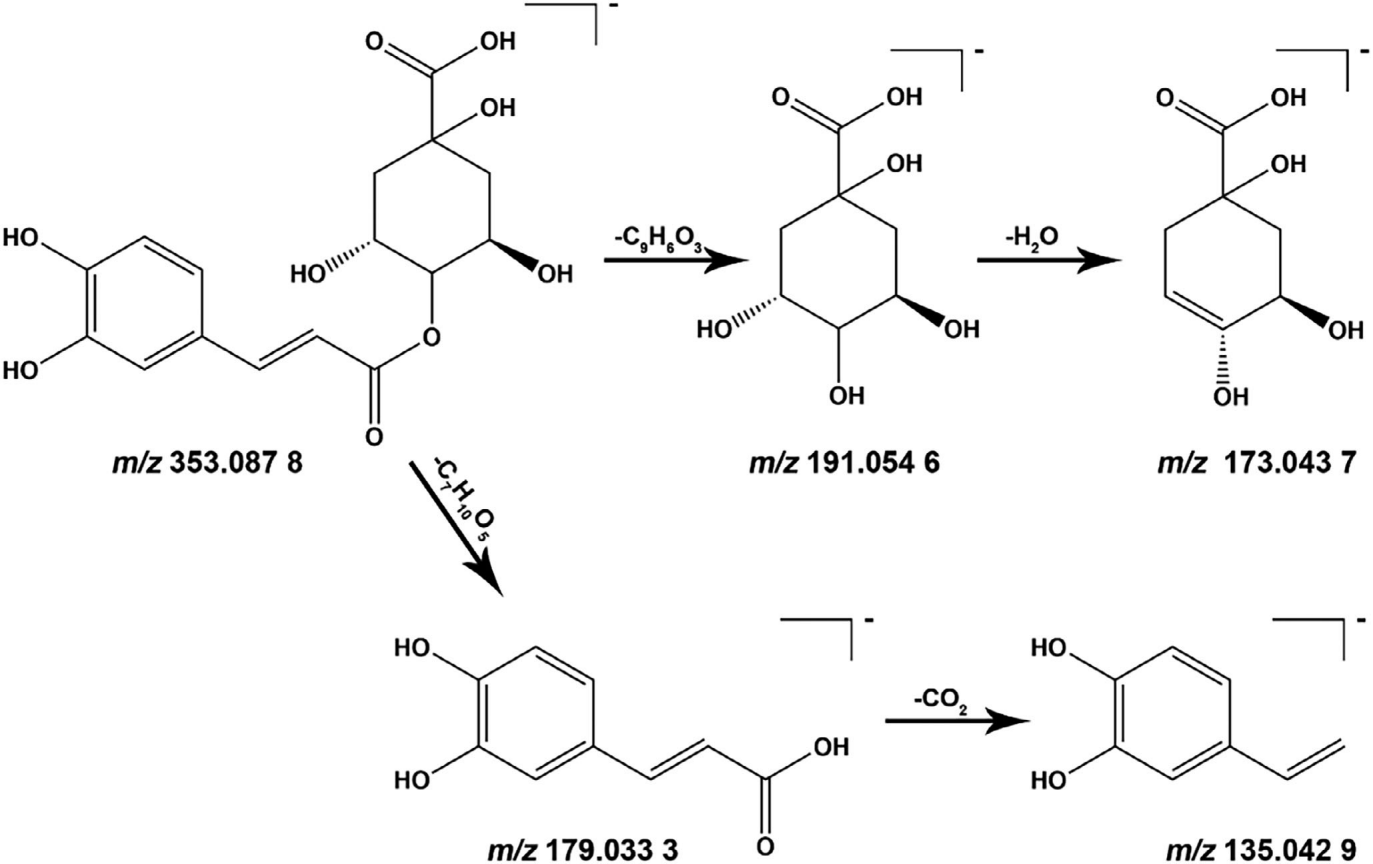
Fragmentation pathways of cryptochlorogenic acid.

For example, Compound 21 exhibited a quasi‐molecular ion at *m/z* 353.0877 [M − H]^−^. Its molecular formula was determined as C_16_H_18_O_9_. On the basis of fragment ions at *m/z* 191.0546, 179.0333, 173.0437 and 135.0429, the presence of caffeic acid and quinic acid structural units was suggested. Database retrieval combined with literature comparison indicated this compound as cryptochlorogenic acid [12, 15, 16, 17], with its fragmentation pathways illustrated in Figure 4.

**FIGURE 5:**
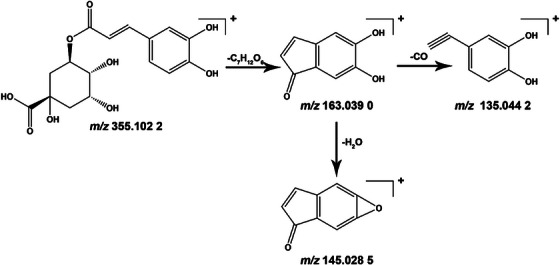
Fragmentation pathways of chlorogenic acid.

For instance, Compound 22 exhibits a quasi‐molecular ion at *m/z* 355.1022 [M + H]^+^. The fitted molecular formula is C_16_H_18_O_9_. Under positive ion mode collision‐induced dissociation, we postulate that its quasi‐molecular ion initially loses C_7_H_12_O_6_ to yield a fragment ion at *m/z* 163.0390. This fragment further loses one H_2_O molecule to form *m/z* 145.0285 or loses one CO molecule to generate *m/z* 135.0442. Through database retrieval combined with literature comparison, this compound is tentatively identified as chlorogenic acid [18], with its fragmentation pathways illustrated in Figure 5.

#### 3.2.4 Isoflavonoids

A total of 10 isoflavonoids were identified in YG samples, including 4 isoflavone glycosides. Flavonoid glycosides typically lose all sugar moieties in mass spectrometry, generating high‐abundance aglycone ions.

**FIGURE 6:**
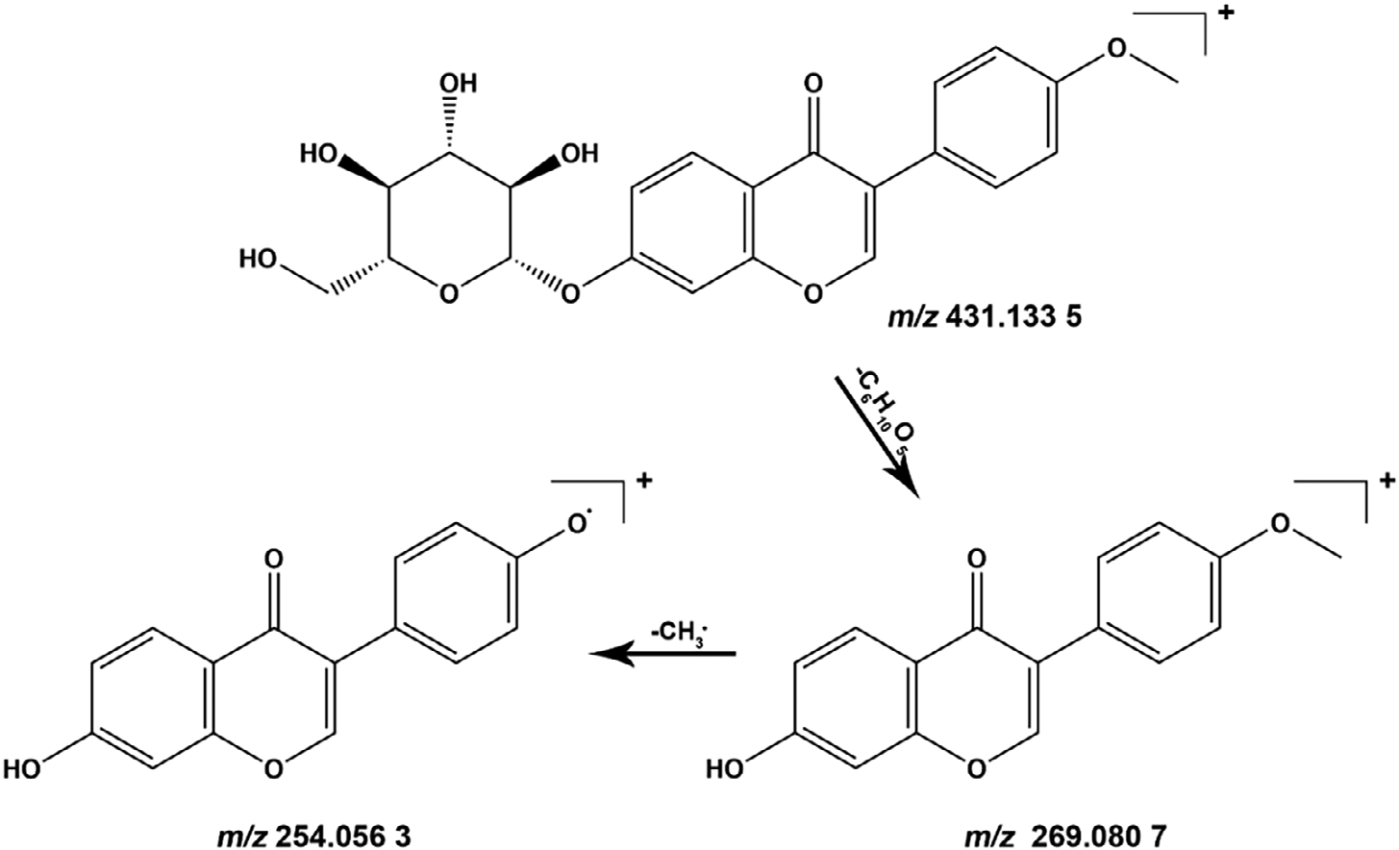
Fragmentation pathways of ononin.

Taking Compound 81 as an example, its quasi‐molecular ion appeared at *m/z* 431.1335 [M + H]^+^ with a molecular formula of C_22_H_22_O_9_. Under positive ion mode collision‐induced dissociation, it is speculated that the quasi‐molecular ion initially loses one glucose molecule to produce the characteristic fragment ion at *m/z* 269.0807 [M + H–C_6_H_10_O_5_]^+^. Subsequently, it undergoes methyl radical elimination to form the fragment ion at *m/z* 254.0563 [M + H–C_6_H_10_O_5_–CH_3_]^+^. On the basis of the database retrieval and literature comparison, this compound was inferred to be ononin [19, 20], and its fragmentation pathways are shown in Figure 6.

#### 3.2.5 Analysis of Blood‐Absorbed Prototype Components in YG

**FIGURE 7:**
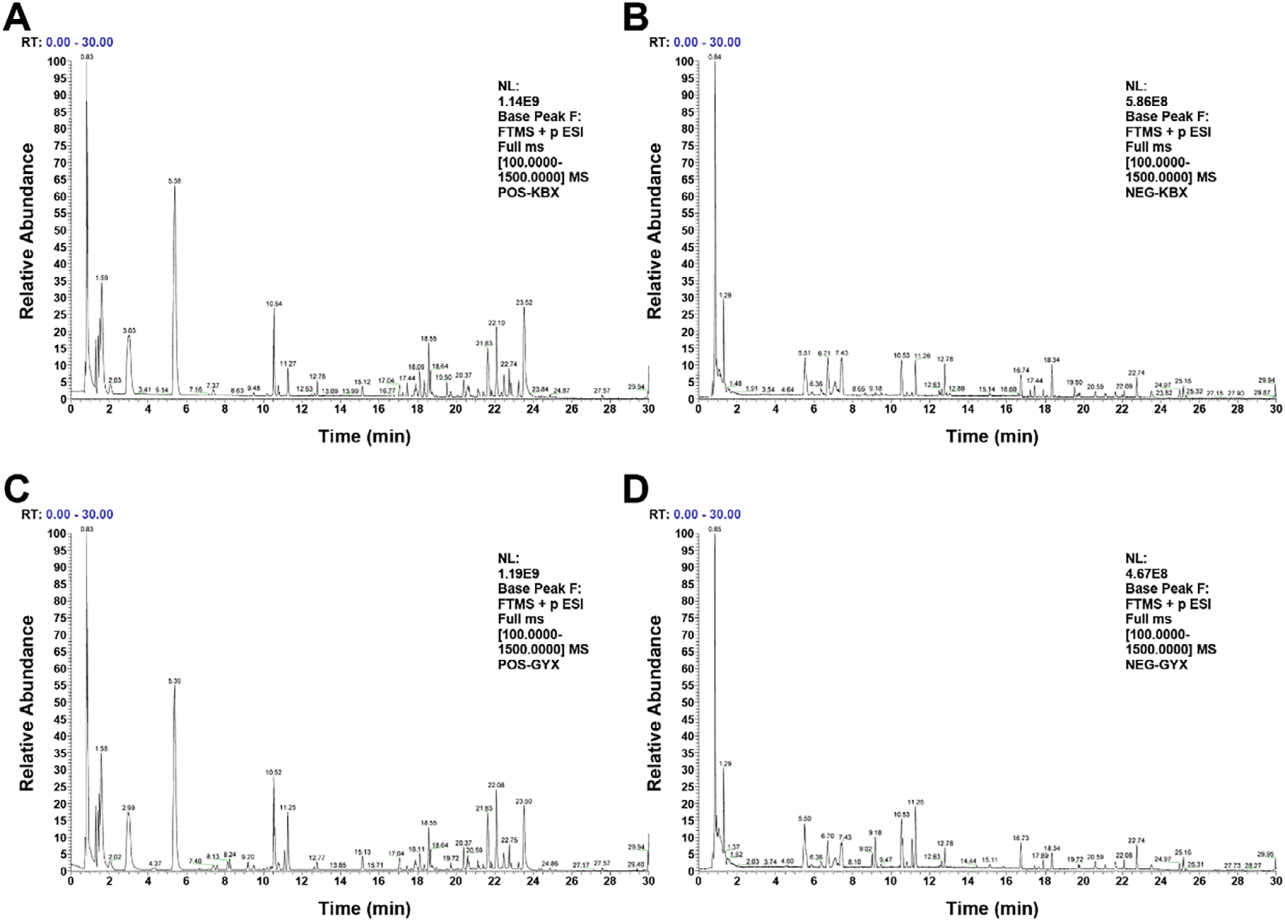
Base peak ions (BPI) chromatograms: (A) blank serum sample in positive ion mode; (B) blank serum sample in negative ion mode; (C) drug‐containing serum sample in positive ion mode; and (D) drug‐containing serum sample in negative ion mode.

**FIGURE 8:**
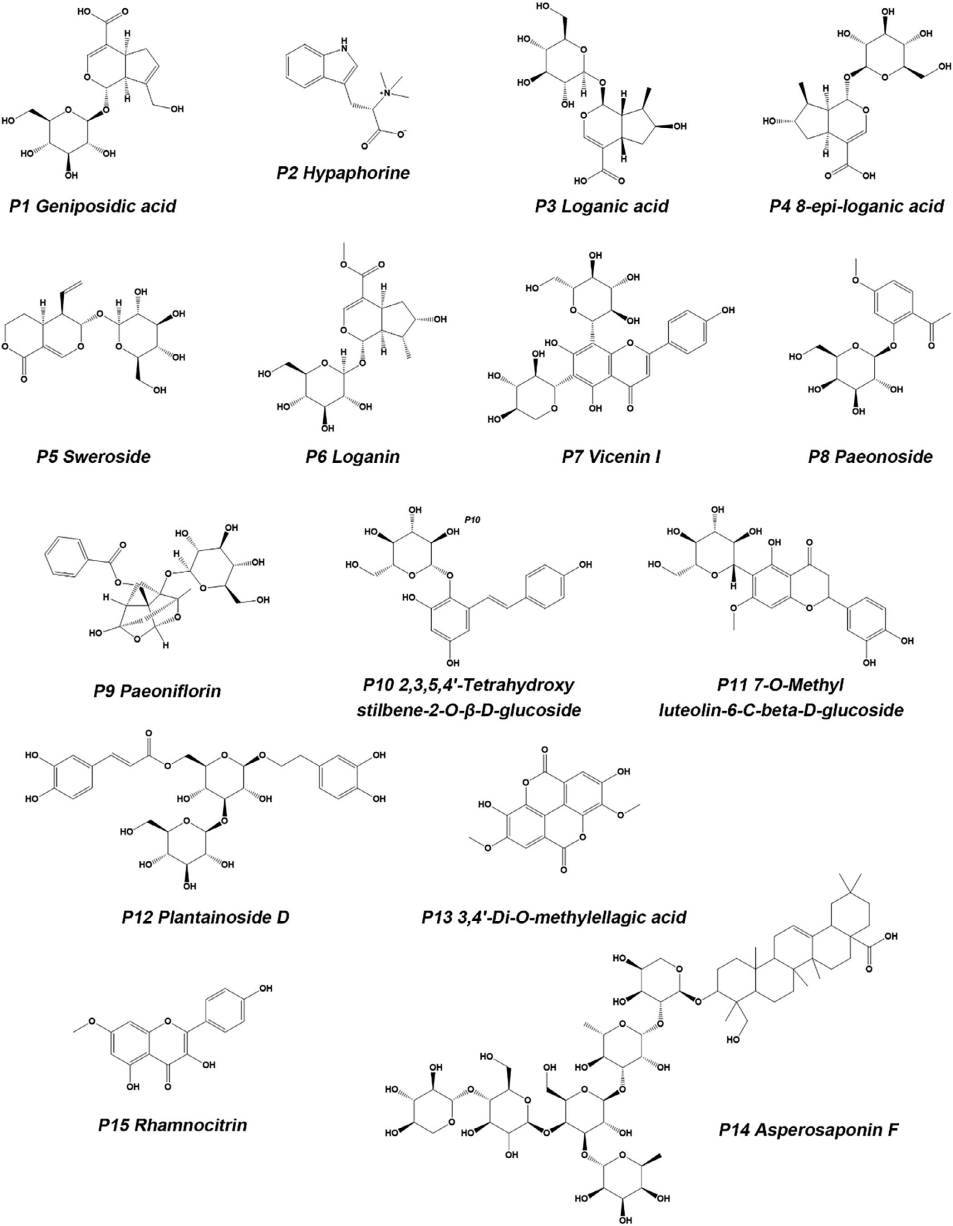
Structural formula of blood‐absorbed prototype components in YG.

As shown in Figure 7, by comparing the BPI chromatograms of YG sample, drug‐containing serum sample and blank serum sample, compounds present in both YG and drug‐containing serum samples but absent in blank serum were identified as blood‐absorbed prototype components, and compounds present in drug‐containing serum but absent in both YG and blank serum samples were inferred to be blood‐absorbed metabolites. After combining with primary and secondary mass spectrometry analysis and comparing data with Table 1 and the database, a total of 15 blood‐absorbed prototype components were identified. The results are presented in Table 2, with their chemical structures shown in Figure 8.

**TABLE 2:** Blood‐absorbed prototype components in rats after gavage administration of Youjing Granule (YG).

No.	Identification	*t*_R_ (min)	Formula	Adducts	Experimental *m*/*z*	Theoretical *m*/*z*	Error (ppm)	Source
P1	Geniposidic acid	5.09	C_16_H_22_O_10_	[M − H]^−^	373.1137	373.1140	−0.80	CQZ
P2	Hypaphorine	6.78	C_14_H_18_N_2_O_2_	[M + H]^+^	247.1442	247.1441	0.42	—
P3	Loganic acid	7.15	C_16_H_24_O_10_	[M − H]^−^	375.1293	375.1297	−0.96	XD
P4	8‐Epi‐loganic acid	7.22	C_16_H_24_O_10_	[M + H]^+^	377.1441	377.1442	−0.23	CQZ
P5	Sweroside	8.98	C_16_H_22_O_9_	[M + H]^+^	359.1338	359.1337	0.34	XD, MDP, BJ
P6	Loganin	9.06	C_17_H_26_O_10_	[M + NH_4_]^+^	408.1868	408.1870	−0.42	XD
P7	Vicenin I	9.20	C_26_H_28_O_14_	[M + H]^+^	565.1549	565.1552	−0.53	SYZ, CBZ, BMG, SCP, HQ
P8	Paeonoside	9.20	C_15_H_20_O_8_	[M + FA‐H]^+^	373.1137	373.1140	−0.79	MDP
P9	Paeoniflorin	9.88	C_23_H_28_O_11_	[M + NH_4_]^+^	498.1976	498.1975	0.09	MDP, FL
P10	2,3,5,4′‐Tetrahydroxy stilbene‐2‐*Ο*‐β‐d‐glucoside	11.59	C_20_H_22_O_9_	[M + NH_4_]^+^	424.1606	424.1608	−0.41	ZSW
P11	7‐*O*‐Methyl luteolin‐6‐C‐β‐d‐glucoside	12.13	C_22_H_24_O_11_	[M − H]^−^	463.1246	463.1246	0.09	SYZ
P12	Plantainoside D	12.79	C_29_H_36_O_16_	[M − H]^−^	639.1930	639.1931	−0.05	CQZ
P13	3,4′‐Di‐*O*‐methylellagic acid	15.69	C_16_H_10_O_8_	[M − H]^−^	329.0303	329.0303	0.09	SYZ
P14	Asperosaponin F	18.94	C_64_H_104_O_30_	[M − H]^−^	1351.6544	1351.6540	0.35	XD
P15	Rhamnocitrin	19.18	C_16_H_12_O_6_	[M + H]^+^	301.0702	301.0707	−1.67	SYZ, HQ

Abbreviations: BJ, Bijie; BMG, Baimaogen; CBZ, ChaobaizhuCQZ, Cheqianzi; FL, Fulin; HQ, Huangqi; MDP, Mudanpi; ML, Muli; SCP, Shichangpu; SYZ, Shayuanzi; TSZ, Tusizi; XD, Xuduan; ZJC, Zaojiaoci; ZSW, Zhiheshouwu.

CQZ contributes three constituents: geniposidic acid, 8‐epi‐loganic acid and plantainoside D; DX provides four constituents: loganic acid, sweroside, loganin and asperosaponin F; three constituents originate from MDP: sweroside, paeonoside and paeoniflorin; four components are derived from SYZ, including vicenin I, 7‐*O*‐methyl luteolin‐6‐C‐β‐d‐glucoside, 3,4′‐*O*‐dimethyl ellagic acid and rhamnocitrin; two components originate from HQ: vicenin I and rhamnocitrin. Additionally, sweroside is present in BJ; vicenin I also exists in CBZ, BMG and SCP; paeoniflorin is further found in FL.

**FIGURE 9:**
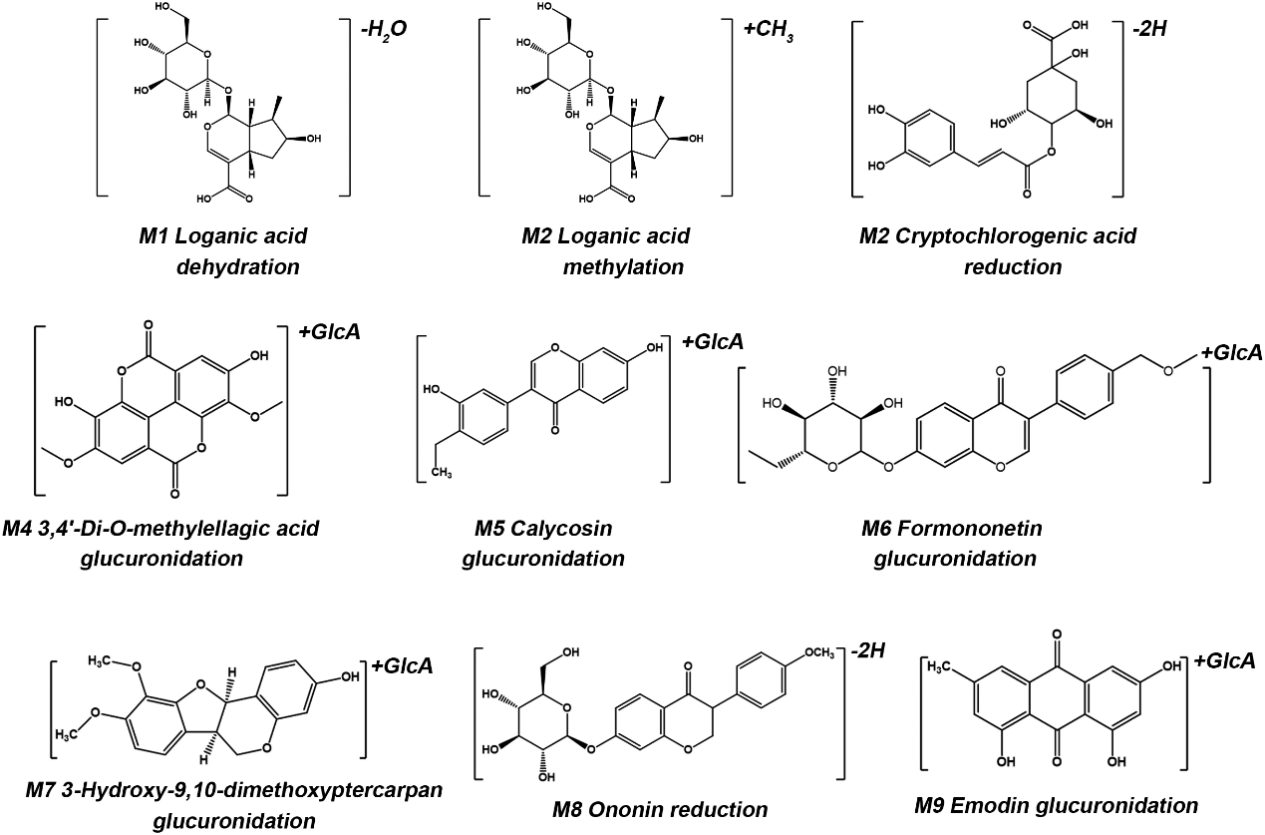
Chemical structural formula of blood‐absorbed metabolites in YG.

By comparing Figure 7 and Table 1, we analysed the potential metabolic pathways of YG and identified metabolites on the basis of mass spectrometric fragmentation patterns. A total of nine metabolites were successfully identified in the drug‐containing serum, with results detailed in Table 3 and Figure 9. Among these, M1, M3 and M8 are Phase I metabolites, whereas M2, M4, M5, M6, M7 and M9 are Phase II metabolites. Taking M6 as an example, this compound forms an [M − H]^−^ quasi‐molecular ion in negative ion mode, with *m/z* 443.0981 [M − H]^−^. The fitted molecular formula is C_22_H_20_O_10_. The prototype compound formononetin generates a quasi‐molecular ion at *m/z* 267.0660 [M − H]^−^ in negative ion mode. The mass difference between them is *m/z* 176.0321, consistent with the molecular weight shift characteristic of glucuronic acid conjugation reactions [21]. Subsequently, by comparing characteristic fragment ions of M6 with those of the prototype compound formononetin, we identified M6 as a glucuronidation product of formononetin.

**TABLE 3:** Blood‐absorbed metabolites in rats after gavage administration of Youjing Granules.

No.	*t*_R_ (min)	Formula	Adducts	Experimental *m*/*z*	Theoretica *m*/*z*	Error (ppm)	Identification	Source
M1	8.96	C_16_H_22_O_9_	[M + FA‐H]^−^	403.1242	403.1246	−0.89	Loganic acid dehydration	XD
M2	9.03	C_17_H_26_O_10_	[M + FA‐H]^−^	435.1504	435.1508	−0.91	Loganic acid methylation	XD
M3	9.15	C_16_H_20_O_9_	[M + FA‐H]^−^	401.1086	401.1089	−0.88	Cryptochlorogenic acid reduction	XD, CBZ, BMG, TSZ, SYZ, BJ, CQZ
M4	11.70	C_22_H_18_O_14_	[M + NH_4_]^+^	524.1044	524.1040	0.69	3,4′‐Di‐*O*‐methylellagic acid glucuronidation	SYZ
M5	12.52	C_22_H_20_O_11_	[M − H]^−^	459.0933	459.0933	−0.02	Calycosin glucuronidation	SYZ, HQ
M6	14.20	C_22_H_20_O_10_	[M − H]^−^	443.0981	443.0984	−0.53	Formononetin glucuronidation	SYZ, HQ
M7	15.30	C_23_H_24_O_11_	[M + NH_4_]^+^	494.1661	494.1662	−0.23	3‐Hydroxy‐9,10‐dimethoxyptercarpan glucuronidation	HQ
M8	15.44	C_22_H_24_O_9_	[M + FA‐H]^+^	477.1400	477.1402	−0.41	Ononin reduction	SYZ, BMG, HQ
M9	17.32	C_21_H_18_O_11_	[M − H]^−^	445.0778	445.0776	0.27	Emodin glucuronidation	ZSW, XD, CBZ, SCP

Abbreviations: BJ, Bijie; BMG, Baimaogen; CBZ, ChaobaizhuCQZ, Cheqianzi; FL, Fulin; HQ, Huangqi; MDP, Mudanpi; ML, Muli; SCP, Shichangpu; SYZ, Shayuanzi; TSZ, Tusizi; XD, Xuduan; ZJC, Zaojiaoci; ZSW, Zhiheshouwu.

## 4 Discussion

Currently, infertility affects 8%–12% of couples of childbearing ages, with male factors directly causing or contributing to approximately 50% of these cases [2]. Male infertility patients commonly exhibit clinical characteristics such as low ejaculate volume, reduced sperm concentration or decreased sperm motility, imposing substantial physical and psychological burdens on numerous families. Most TCM scholars recognize deficiency of kidney essence as the pathogenesis of male infertility. Physicians throughout history have consistently regarded tonifying kidney essence as the fundamental therapeutic approach, while concurrently regulating the heart, spleen and lung [5]. YG also adopts a method combining tonifying kidney and resolving dampness with removing stasis and clearing heat. Among the components, SYZ possesses warm nature and sweet flavour, acting on the liver and kidney meridians. It functions to warm and tonify the liver and kidney, reduce urination and alleviate leucorrhoea. Its extract significantly reduces the sperm deformity index in animals with spermatogenic dysfunction, markedly enhances sperm quality in rats, substantially increases serum testosterone levels, decreases follicle‐stimulating hormone and demonstrates a remarkable spermatogenic‐promoting effect [6, 22]. Both CQZ and TSZ are core components for treating asthenospermia. When combined with other ingredients, they demonstrate remarkable efficacy in tonifying the kidney, promoting spermatogenesis and activating sperm vitality, thereby enhancing sperm motility and increasing progressive sperm count [7, 23]. DX possesses a bitter, sweet and pungent taste with a slightly warm nature. It functions to tonify the liver and kidney while strengthening bones and tendons. Studies indicate that DX can counteract the reproductive damage induced by Tripterygium hypoglaucum in male rats [24]. MDP exhibits a slightly cold nature with pungent and bitter flavours and enters the heart, liver and kidney meridians, demonstrating effects in clearing heat, cooling blood, activating blood circulation and dissipating blood stasis. It serves as an essential component in numerous TCM formulations for treating male infertility [5, 23, 25].

As a time‐tested clinical prescription for treating male infertility, YG demonstrates remarkable therapeutic efficacy and are extensively applied in clinical practice. However, their complex chemical composition and unidentified active pharmaceutical ingredients limit further development. Therefore, systematic characterization of their complex chemical profile, along with subsequent identification of the components absorbed into the bloodstream after oral administration, is crucial for elucidating the material basis of their pharmacological effects and advancing modern research into their mechanisms of action. Blood‐absorbed components of TCM are considered potential bioactive constituents. Further analysis of the components from YG that enters systemic circulation holds significant value for identifying pharmacologically active substances and elucidating the mechanisms of action. UHPLC‐Q‐orbitrap‐MS technology, characterized by high resolution, accuracy and separation efficiency, serves as an effective approach for rapid separation and identification of chemical components in complex TCM systems.

The metabolism of reactive oxygen species (ROS) profoundly influences male reproductive capacity, where appropriate levels of ROS facilitate sperm capacitation and acrosome reactions [5]. However, excessive ROS may trigger redox imbalance, induce oxidative stress damage and adversely affect spermatogenesis. Under oxidative stress conditions, endogenously generated damage‐associated molecular patterns (DAMPs) are activated by ROS, stimulating cytokine production and subsequently activating downstream signalling pathways such as nuclear factor‐κ B (NF‐κB) and mitogen‐activated protein kinases (MAPK). This cascade amplifies chemokine expression, recruits additional inflammatory cells and ultimately initiates sterile inflammation [5]. The inflammatory process leads to persistent lipid peroxidation, causing accumulation of O_2_·^−^, which further reacts with unstable hydrogen atoms on polyunsaturated fatty acid, triggering a cascade effect that ultimately reduces sperm plasma membrane fluidity [5].

This experiment employed UHPLC‐Q‐orbitrap‐MS technology to analyse YG and serum samples from rats following YG administration via gavage. Ultimately, 132 chemical components were identified and analysed from YG, with 24 blood‐absorbed components detected in serum samples, including 15 prototype components and 9 metabolites. Metabolic pathways involved Phase I metabolic reactions (dehydration, reduction) and Phase II metabolic reactions (glucuronidation, methylation). Specific results are presented in Tables 2 and 3. Wang et al.’s research demonstrated that the blood‐absorbed prototype component sweroside inhibits the ROS‐mediated NF‐κB/NLRP3 pathway in Ang II‐treated cardiomyocytes by directly binding to CaMKIIδ [26]. Ma et al.’s research indicates that sweroside suppresses aconitine‐induced intracellular ROS generation [27]. Additionally, sweroside exhibits anti‐inflammatory [28], antioxidant [29] and apoptosis‐inhibiting properties [30]. Geniposidic acid effectively reduces ROS levels, inhibits JNK activation and suppresses apoptosis, demonstrating its key role in regulating ROS/JNK/NLRP3 signalling in cell death [31]. Furthermore, geniposidic acid exerts anti‐aging effects through antioxidant stress response and autophagy induction [32]. Hypaphorine demonstrates anti‐inflammatory effects in sepsis‐induced acute lung injury by modulating the DUSP1/p38/JNK signalling pathway [33]. Hypaphorine also mediates anti‐inflammatory effects in endothelial cells via the AMPK signalling pathway by facilitating interaction between TLR4 and PPARγ [34].

In conclusion, this study employed UHPLC‐Q‐orbitrap‐MS technology to characterize the chemical components, blood‐absorbed prototype components and metabolites of YG, laying the theoretical groundwork for further YG research.

## Author Contributions

**Mingxin Guo**: writing – original draft, writing – review and editing. **Jiaqi Zeng**: writing – original draft, writing – review and editing. **Xuping Jiang**: formal analysis. **Wenjiao Zhu**: formal analysis, investigation. **Zhian Tang**: conceptualization, funding acquisition, writing – review and editing. **Tieliang Ma**: conceptualization, funding acquisition, writing – review and editing. All authors contributed to revision and approved the final version for publication.

## Funding

This work was funded by the National Natural Science Foundation of China (Grants 82404987 and 82505411), the Top Talent Support Program for Young and Middle‐aged People of the Wuxi Health Committee (Grant BJ2020105) and the foundation of the Wuxi Administration of Traditional Chinese Medicine (Grants 2023‐ZYYB29 and 2023‐ZYYB31).

## Conflicts of Interest

The authors declare no conflicts of interest.

## Data Availability

Data sharing is not applicable to this article as no data were created or analysed in this study.
